# The basal function of teleost prolactin as a key regulator on ion uptake identified with zebrafish knockout models

**DOI:** 10.1038/srep18597

**Published:** 2016-01-04

**Authors:** Yuqin Shu, Qiyong Lou, Ziru Dai, Xiangyan Dai, Jiangyan He, Wei Hu, Zhan Yin

**Affiliations:** 1 Key Laboratory of Aquatic Biodiversity and Conservation of the Chinese Academy of Sciences, Institute of Hydrobiology, Chinese Academy of Sciences, Wuhan, Hubei, 430072, China; 2 University of the Chinese Academy of Sciences, Beijing, China; 3 Key Laboratory of Molecular Biophysics of Minstry of Education, College of Life Science and Technology, Center for Human Genome Research, Huazhong University of Science and Technology, Wuhan, Hubei 430074, P. R. China

## Abstract

Prolactin (PRL) is an anterior pituitary hormone with a broad range of functions. Its ability to stimulate lactogenesis, maternal behavior, growth and development, osmoregulation, and epithelial ion transport has been reported in many vertebrates. In our present study, we have targeted the zebrafish *prl* locus via transcription activator-like effector nucleases (TALENs). Two independent targeted mutant lines with premature termination of the putative sequence of PRL peptides were generated. All *prl*-deficien*t* zebrafish progeny died at 6–16 days post-fertilization stage (dpf) in egg water. However, the *prl*-deficient larvae thrived and survived through adulthood in brackish water (5175 mg/L ocean salts), without obvious defects in somatic growth or reproduction. When raised in egg water, the expression levels of certain key Na^+^/Cl^−^ cotransporters in the gills and Na^+^/K^+^-ATPase subunits, Na^+^/H^+^ exchangers and Na^+^/Cl^−^ transporters in the pronephros of *prl*-deficient larvae were down-regulated at 5 dpf, which caused Na^+^/K^+^/Cl^−^ uptake defects in the mutant fish at 6 dpf. Our present results demonstrate that the primary function of zebrafish *prl* is osmoregulation via governing the uptake and homeostasis of Na^+^, K^+^ and Cl^−^. Our study provides valuable evidence to understand the mechanisms of PRL function better through both phylogenetic and physiological perspectives.

Prolactin (PRL) was first discovered biochemically around 1930 by Oscar Riddle in pigeons, a non-mammalian animal^1^. However, PRL is best known for its role in enabling female mammals to produce milk, and has more diverse biological functions than all other vertebrate pituitary hormones. PRL has been suggested to play essential roles in metabolism, regulation of the immune system, osmoregulation, and pancreatic development. In rodents, PRL and PRL-like genes are also thought to be involved in placental development. PRLR knockout mice clearly demonstrate an essential role of PRL in mammary gland development, lactation and embryonic development, while PRL transgenic mice developed mammary carcinomas through activation of PRLR^2^,^3^. Apart from its role in mammary gland development and lactation, its functions in reproductive processes differ markedly from one species to another^4^. The lack of a single common physiological effect in non-mammals has provoked the question of whether a basal function of PRL exists in vertebrates^5^. Most current knowledge of PRL function has been dominated by studies of rodent models, especially genetic mouse models. However, it might be useful to step back and explore the pre-mammalian evolution of PRL to understand the nature of PRL functioning better.

With more than 25,000 species, fish are the most diverse group of vertebrates, and offer a valuable comparative system for the study of endocrine activities. There is a vast body of literature regarding the broad spectrum of PRL actions in teleosts, encompassing growth and development, metabolism, behavior, reproduction, immunoregulation, and osmoregulation^6^,^7^. A classic surgical “ablation” and “replacement” experiment on killifish (*Fundulus heteroclitus*) performed by Pickford in 1959 demonstrated the essential requirement of PRL for this euryhaline species to live in freshwater^8^. PRL was later shown to regulate the ion uptake as well as water permeability of osmoregulatory surfaces of the whole fish in many euryhaline species by acting on the epithelia system such as the gills, kidneys, intestines, and urinary bladder^9^,^10^,^11^.

Zebrafish represent a recent and well-utilized model for developmental biology. As stenohaline cyprinids, zebrafish inhabit a hypotonic freshwater environment and have recently emerged as a powerful teleost model to study osmoregulation. Four types of ionocytes expressing different sets of ion transporters have been identified in zebrafish: H^+^-ATPase-rich (HR), Na^+^-K^+^-ATPase-rich (NaR), Na^+^-Cl^−^ cotransporter (NCC), and K^+^-secreting (KS) cells. These ionocytes perform trans-epithelial H^+^ secretion/Na^+^ uptake/NH_4_^+^ excretion, Ca^2+^ uptake, Na^+^/Cl^−^ uptake, and K^+^ secretion, respectively^12^. As in many other teleosts, an additional copy of the PRL gene (*prl2*) has been identified in zebrafish, which arose from whole genome duplication in these vertebrates after their divergence from jawless fish. However, zebrafish PRL2 was shown to be expressed highly in the eye and brain but not in the pituitary of zebrafish. It has been suggested to be primarily involved in retinal development^13^. In contrast, PRL is common to all vertebrates, is mainly expressed in the pituitary gland, and is generally referred to simply as PRL^12^.

Recently, through intraperitoneal injection of ovine PRL or addition of a human PRL receptor antagonist (Δ-9-G129R-hPRL) to zebrafish or cultured zebrafish gill filaments, Braves *et al.* (2013) have demonstrated that zebrafish PRL can positively regulate NCC expression in the zebrafish gill both *in vivo* and in culture^14^.

In our present study, to understand the potentially basal function of PRL better, the zebrafish *prl* locus was targeted via transcription activator-like effector nucleases (TALENs) to generate putative truncated PRL peptides. All *prl*-deficient zebrafish progenies died at 6–16 days post-fertilization (dpf) stage in regular egg medium but thrived in hypertonic (brackish) water, showing no obvious defects in growth or reproduction. Decreased expression of ion transporter genes and Na^+^ and K^+^ body contents of 5 dpf *prl*-deficient fish kept in regular egg water indicated that depletion of PRL heavily impaired absorption of Na^+^/K^+^/Cl^−^ and secretion of H^+^/NH_4_^+^ in zebrafish, strongly suggesting that the function of PRL in freshwater fish primarily involves osmoregulation. Our study provides valuable evidence for a more thorough understanding of the mechanisms of PRL function through both phylogenetic and physiological perspectives.

## Results

### *Prl* gene knockout in zebrafish

**Figure 1:**
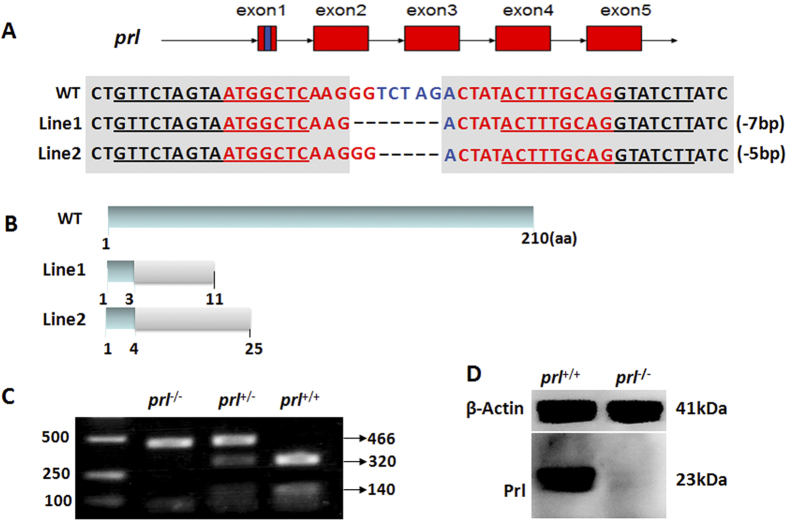
Inactivation of the zebrafish *prl* gene. (**A**) Schematic representation of wild-type (*prl*^+/+^, top) and two lines of TALEN targeted *prl* alleles (center and bottom). Exons are represented by red boxes with the targeted region in exon 1 shown as a blue box. TALEN arms are marked by an underline in the targeted region of the wild-type locus. (**B**) Schematic representation of the putative peptide of wild-type PRL (top) and the two mutated PRL peptides from the targeted alleles (center and bottom). (**C**) Genomic DNA PCR assay of 5 dpf larvae resulting from a cross between *prl* heterozygotes. (**D**) Western blot of the pituitary samples from wild-type (left) and *prl*-deficient (right) adults at 100 dpf. WT, wild-type; TALEN, transcription activator-like effector nuclease; PCR, polymerase chain reaction; Dpf, days post-fertilization.

*Prl*-depletion zebrafish were generated using the TALENs gene targeting technique. The target sequences were designed based on the sequence of *prl* provided in NCBI, against the first exon of *prl* with the addition of an *Xba*I restriction site for confirmation of the mutation. Both arms of the TALEN were 16 bp in length (Fig. 1A). Two independent mutant lines were obtained with 5 or 7 bp deletions in the spacer region in the first exon of *prl* (Fig. 1A). The putative *prl* protein of wild-type zebrafish contains 210 amino acids, while the two mutant lines only retained 3 or 4 correct amino acids, respectively. The translation of both putative transcripts from the two mutant lines exhibited premature stops at the 11th or 25th amino acid residues (Fig. 1B). Identification of mutants was detected by genotyping. Amplified fragments of 466 bp containing unmodified targeted sites could be screened out following *Xba*1 digestion: the digested fragments from wild-type samples showed two bands of 320 and 140 bp, whereas those from the homozygous mutants showed only an undigested 466 bp fragment, and those from heterozygous offspring exhibited three bands because of partial *Xba*1 digestion (Fig. 1C). In addition, PRL levels were confirmed to be deficient in the mutants by western blotting using pituitary extracts (Fig. 1D).

### Survival of *prl* knockout zebrafish is restricted to brackish water

**Figure 2:**
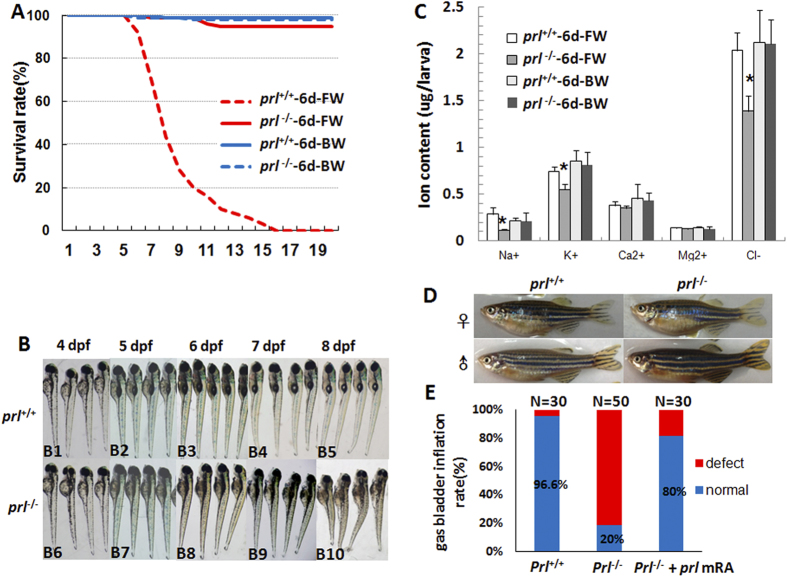
*Prl*-deficient zebrafish can survive only in brackish water. (**A**) The curve of survival rates of wild-type and *prl*-deficient larvae in regular zebrafish egg water and system water (FW, 60–175 mg/L IOS) or brackish water (BW, 5060–5175 mg/L IOS) (n = 100). (**B**) The general morphological observations of wild-type larvae (B1-B5) and *prl*-deficient larvae (B6-B10) during 4–8 dpf. The *prl*-deficient larvae exhibit defects in swim bladder inflation after 5 dpf (B8-B10), curved bodies at 6 dpf (B8), and obvious edema at 7–8 dpf (B9, B10). (**C**) Major ion contents were measured with wild-type larvae and *prl*-deficient larvae at 6 dpf cultured in FW and BW. Cation contents were assessed by the inductively coupled plasma-optical emission spectroscopy (ICP-OES) method and chloride assessed by chloride colorimetric assay kit. *significant difference (P < 0.05). At least twenty embryos were pooled as a sample for ion content analysis. (**D**) Representative healthy wild-type (left) and *prl*-deficient (right) adults cultured in brackish water at 100 dpf. IOS, Instant Ocean salts; dpf, days post-fertilization. E) Assessment of synthesized wild-type *prl* mRNA injection based on statistics of gas bladders inflated rates of wild-type larvae, *prl*-deficient larvae and *prl*-deficient larvae injected with *prl* mRNA at 5 dpf.

When *prl*-deficient larvae were raised in FW (fresh water, salinity: 60–175 mg/L), they began to die at 6 dpf and died out before 20 dpf, whereas the wild-type larvae could survive well (Fig. 2A). However, the *prl*-deficient zebrafish were able to survive in the hypertonic water following additional 5 g IOS (Instant Ocean salts, Coral reef salt, Sunnyvale, CA, Germany) per liter of system water (Fig. 2A), which is equivalent to BW (brackish water, salinity : 5060–5175 mg/L) with its salinity of 30 times higher than FW. In FW, based on our morphological observation of early developmental processes, no obvious defects could be found in the *prl*-deficient larvae prior to 4 dpf (Fig. 2B). After the 5 dpf stage, defects including failure of inflation of the swim bladder, hydrocardia, edema, and curly body were observed in most of the *prl*-deficient larvae. Most were also observed to be lying on their sides on the bottom of the tank with fewer xanthophores (greenish color on the dorsal parts of larvae head region). The defective conditions of the larvae continuously worsened over time, until mortalities in the *prl*-deficient fish with significantly shortened and swollen bodies compared with those of the wild-type control fish began to be observed (Fig. 2B). The phenotypes of the *prl*-deficient larvae were consistent with those previously reported in *prl*-morphants^15^. Additionally, the *prl*-deficient larvae were found to have significantly decreased levels of Na^+^, K^+^ and Cl^−^ but normal levels of calcium and magnesium at 6 dpf, and the decreased ions could recover to normal levels as wild-type larvae when sustained in brackish water (Fig. 2C). These results demonstrated that *prl* may regulate Na^+^, K^+^ and Cl^−^ to carry out its function as a freshwater survival factor. Strikingly, the *prl*-deficient fish could survive in BW without any obvious defect as well as their wild-type control siblings. Both female and male *prl*-deficient fish could be raised to adulthood with no significant differences in body weight and fecundity compared with wild-type control fish also kept in BW (Fig. 2D). These results demonstrated that *prl* is essential for zebrafish larvae to survive in FW, but not essential to growth or fecundity, implying a critical osmoregulatory function of zebrafish *prl*. To confirm the genes-specific effects in the *prl*-deficient fish, wild-type *prl*-mRNA has been synthesized and injected into 1–2 cell stage *prl*-deficient embryos. A significant increase of the rate of the inflated gas bladders of *prl*-deficient larvae at 5 dpf stage has been observed (Fig. 2E).

### *Prl*-depletion down-regulates specific makers for HR cells in the pronephric duct (PD) and NCC cells in the gill epithelium

**Figure 3:**
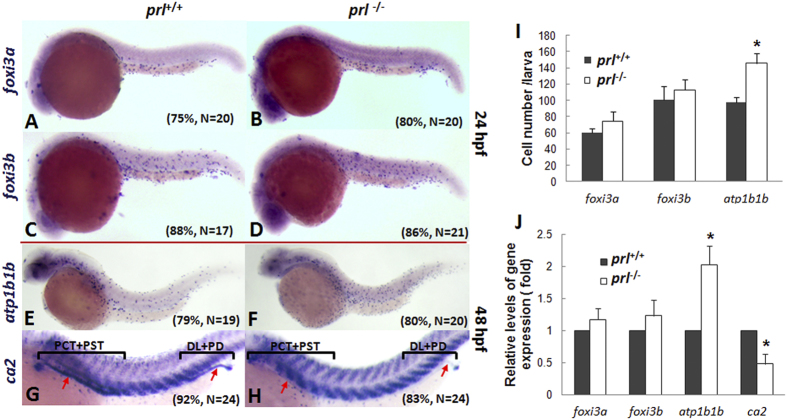
Expression patterns of ionocyte progenitor markers. (**A–H**) Expression patterns of *foxi3a* (A,B) *forkhead box I3a*, an early marker for HR cells) and *foxi3b* (**C,D**) *forkhead box I3b*, an early marker for NaR cells) in wild-type (left) and *prl*-deficient (right) larvae at 24 hpf and *ca2* (**E,F**) *carbonic anhydrase II*, a marker for HR cells) and *atp1b1b* (**G,H**) *ATPase*, Na^+^/K^+^ transporting, beta 1b polypeptide, a marker for NaR cells) in wild-type (left) and *prl*-deficient (right) larvae at 48 hpf. (**I**) counts of *foxi3a, foxi3b* and *atp1b1b* expression cells based on *in situ* results. (**J**) Expression levels of *foxi3a* and *foxi3b* (at 24 hpf), *ca2* and *atp1b1b* (at 48 hpf) in total tissue from wild-type and *prl*-deficient larvae in regular zebrafish egg water via qRT-PCR assay. *significant difference (P < 0.05). Arrows, pronephric ducts; The qRT-PCR result shown here is the representative of the results obtained in two separate experiments. For *in situ* hybridization results, at least 17 embryos/genotype were analyzed in two separated experiments. For cell counts, 8 embryos were analyzed in two groups (N = 8).

For fish to survive in FW environments, both water gain and ion loss across the surface of the epithelium must be tightly regulated. It has been suggested that the tight junction complexes composed of occludins, claudin family proteins, and aquaporin family proteins (AQPs) are crucial for this function^16^,^17^. However, no significantly alterations in the transcription levels of occludins, claudin family protein genes, or aquaporin family protein genes were observed in *prl*-deficient larvae compared to those of control fish (Supplementary Fig. S1). Additionally, mannitol added water acclimation experiment failed to rescue *prl*-defficient larvae though it was in the same osmolarity as the BW (Supplementary Fig. S1). To explore the possible defects in the expression patterns of the ionoregulatory genes in the *prl*-deficient zebrafish, we first observed the developmental process of the ionocyte progenitors. Ionocyte progenitor cells were assayed using forkhead box I3a (*foxi3a*) and -*I3b* (*foxi3b*), which are early makers for the ionocyte progenitors of NaR and HR cells^18^,^19^. No significant differences between the expression patterns of *foxi3a* and *foxi3b* of the *prl*-deficient fish and wild-type controls were observed at the 24 hours post-fertilization (hpf) stage via whole mount *in situ* (Fig. 3A–D). The progress of the ionocyte progenitors were then assayed with *ATPase, Na*^*+*^*/K*^*+*^ *transporting, beta 1b polypeptide* (*atp1b1b*) and *carbonic anhydrase II* (*ca2*), respective markers for the progenitors of NaR or HR cells at a later stage. The increased levels of *atp1b1b* expression in *prl*-deficient fish were observed compared to wild-type control fish at 48 hpf (Fig. 3E,F), while deficiencies of *ca2* expression in the proximal convoluted tubules (PCT), proximal straight tubules (PST), and distal late (DL) and PDs of the pronephros in *prl*-deficient larvae were seen at the 48 hpf stage (Fig. 3G,H). Based on the scatted dot expression patterns of *foxi3a, foxi3b* and *atp1b1b* shown with the *in situ* hybridizations, the levels of the positive cells have been counted with the positive cell number counting. A significantly increased expression levels of *atp1b1b* in the *prl*-deficient fish compared to the control fish has been confirmed via the cell counting (Fig. 3I). The expression levels of the *foxi3a, foxi3b, atp1b1b* and *ca2* assayed with real time PCR also support the hybridization results with increased expression of *atp1b1b* but decreased expression of *ca2* in *prl*-deficient larvae compared with those in control fish at 48 hpf (Fig. 3J).

**Figure 4:**
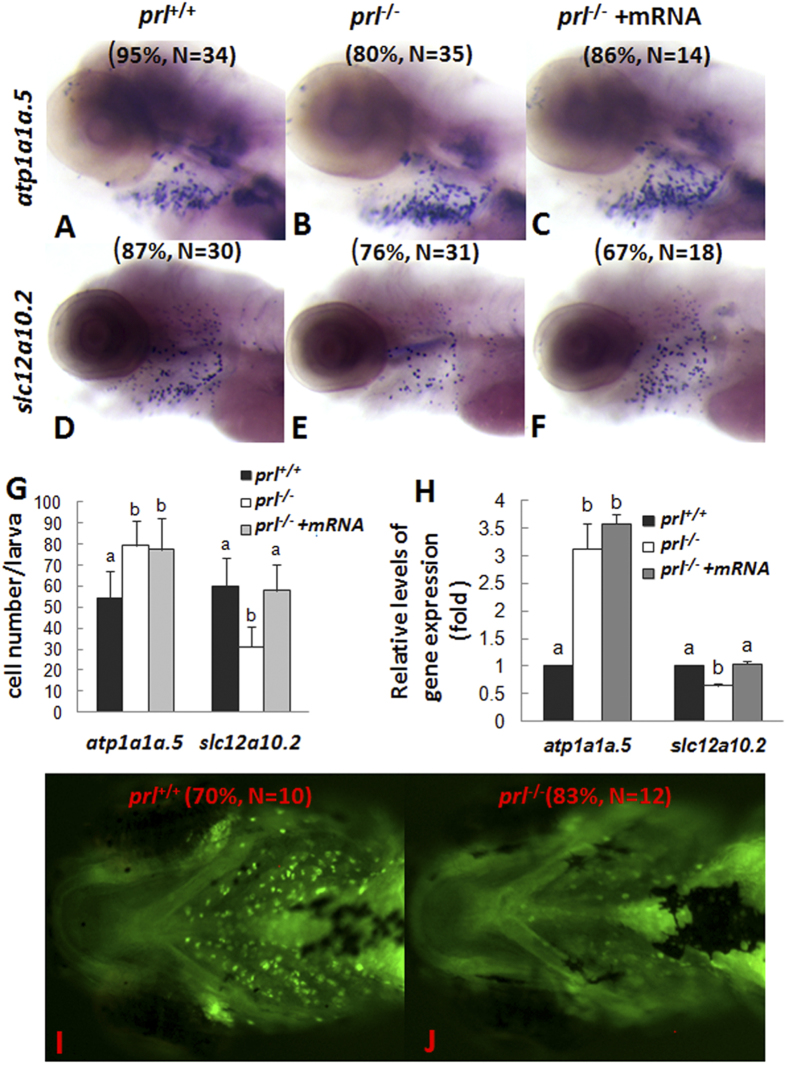
Expression patterns of ion transporters and sodium accumulation in gills of *prl*-deficient larvae. (**A–F**) Whole mount *in situ* hybridization assay of *solute carrier family 12, member 10, tandem duplicate 2* (*slc12a10.2*) and *ATPase, Na/K transporting, α 1a polypeptide, tandem duplicate 5* (*atp1a1a.5*) expression in gills of wild-type larvae (**A,D**), *prl*-deficient larvae (**B,E**) and *prl* -deficient larvae injected with *prl* mRNA (**C,F**) at 5 dpf in regular zebrafish egg water. (**G**) counts of *atp1a1a.5* and *slc12a10.2* expression cells in gills based on *in situ* results. (**H**) Expression levels of *atp1a1a.5* and *slc12a10.2* in head tissue from wild-type, *prl*-deficient larvae and *prl*-deficient larvae injected with *prl* mRNA at 5 dpf in regular zebrafish egg water via qRT-PCR assay. (**I,J**) Sodium green staining in gills of living larvae of wild-type (**I**) and *prl*-deficient larvae (**J**) at 6 dpf in regular zebrafish egg water (dorsal views). (**a,b**) different letters in two group mean significant difference (P < 0.05). The qRT-PCR result shown here is the representative of the results obtained in two separate experiments. For *in situ* hybridization results, at least 12 embryos/genotype were analyzed in two separated experiments. For cell counts, 8 embryos were analyzed in two groups (N = 8).

*Solute carrier family 12, member 10, tandem duplicate 2* (*slc12a10.2*) is a Na^+^/K^+^/Cl^−^ cotransporter in NCC cells and has been proven to play major roles in Cl^−^ and Na^+^ absorption^20^,^21^,^22^. On the other hand, *atp1a1a.5* is a Na^+^- K^+^ exchanger on the basolateral membrane of HR ionocytes that provides a driving force for other gated ion entry^23^,^24^. At the 5 dpf stage, decreased expression levels of *slc12a10.2* (an NCC marker) but increased expression levels of *atp1a1a.5* (*NKA.5*, an HR marker) were observed in gills of *prl*-deficient larvae via whole mount *in situ* (Fig. 4B,E). Both decreased expression levels of *slc12a10.2* and increased expression levels of *atp1a1a.5* were also reflected by cell counting (Fig. 4G) and real time PCR assays (Fig. 4H). Expression of *slc12a10.2* could recover to normal in *prl*-deficient larvae at 5 dpf after synthesized wild-type *prl* mRNA injection (Fig. 4F,H). Notably, although the enhanced levels of *atp1a1a.5* expression were seen, the decreased contents of sodium in gills of *prl*-deficient larvae compared with those of control fish have been observed via sodium green staining in live larvae (Fig. 4I,J), which suggesting that the gill HR cells might not be responsible for the ion loss in the live *prl*-deficient larvae.

**Figure 5:**
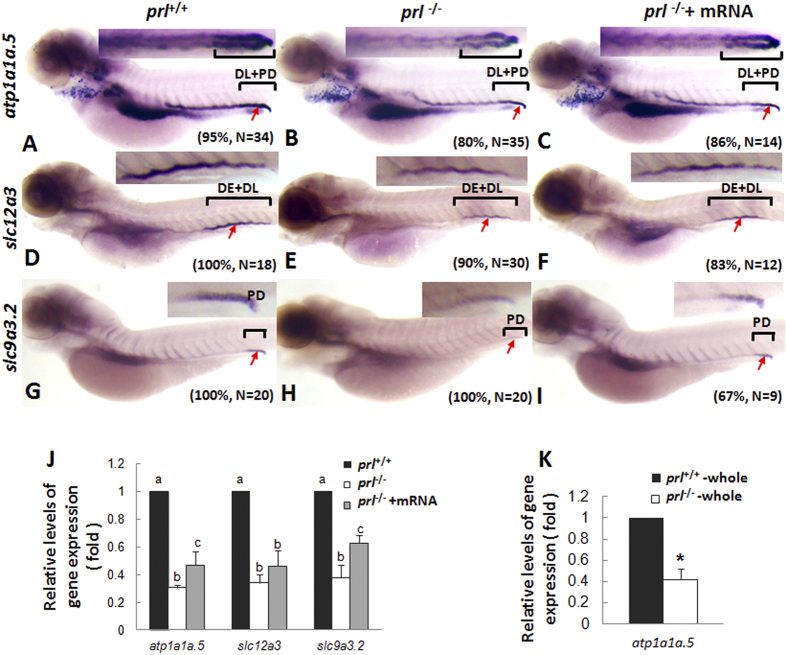
Expression patterns of ion transporters in pronephros of *prl*-deficient larvae. (**A–C**) Whole mount *in situ* hybridization assay of *atp1a1a.5* expression in pronephros of wild-type larvae (**A**), *prl*-deficient larvae (**B**) and *prl*-deficient larvae injected with *prl* mRNA (**C**) at 5 dpf in regular zebrafish egg water. (**D–F**) Whole mount *in situ* hybridization assay of *solute carrier family 12, member 3* (*slc12a3*) expression in pronephros of wild-type larvae (**D**), *prl*-deficient larvae (**E**) and *prl*-deficient larvae injected with *prl* mRNA (**F**) at 5 dpf in regular zebrafish egg water. (**G–I**) Whole mount *in situ* hybridization assay of *solute carrier family 9, subfamily A, member 3, tandem duplicate 2* (*slc9a3.2*) expression in pronephros of wild-type larvae (**G**), *prl*-deficient larvae(**H**) and *prl*-deficient larvae injected with *prl* mRNA (**I**) at 5 dpf in regular zebrafish egg water. Insets in A-I: the amplified images of the pronephros. (**J**) Expression levels of *atp1a1a.5, slc12a3*, and *slc9a3.2* in decapitated larvae body samples from wild-type, *prl*-deficient larvae and *prl*-deficient larvae injected with *prl* mRNA at 5 dpf in regular zebrafish egg water assayed via qRT-PCR assay. (**K**) Expression levels of *atp1a1a.5* in total tissue from wild-type and *prl*-deficient larvae at 5 dpf in regular zebrafish egg water assayed via qRT-PCR assay. *significant difference (P < 0.05). (**a–c**) different letters in two group mean significant difference (P < 0.05). Arrows, pronephric ducts; The qRT-PCR result shown here is the representative of the results obtained in two separate experiments. For *in situ* hybridization results, at least 12 embryos/genotype were analyzed in two separated experiments.

Furthermore, decreased expression patterns of *atp1a1a.5, solute carrier family 12, member 3* (*slc12a3*, a Na^+^/Cl^−^ transporter), and *slc9a3.2* (an HR marker) in pronephros of *prl*-deficient larvae compared with those of wild-type control fish were observed at 5 dpf via whole mount *in situ* hybridization, with the levels could be partially recovered with the synthesized wild-type *prl* mRNA injection (Fig. 5A–I). Utilizing real time PCR assays with the decapitated body samples, the decreased expression levels of *atp1a1a.5, slc12a3*, and *slc9a3.2* in pronephros of *prl*-deficient larvae compared with those of wild-type control fish were also confirmed (Fig. 5J). Although enhanced atp1a1a.5 expression was seen in the gill of *prl*-deficient fish (Fig. 4), an overall decreased levels of its expression in *prl*-deficient larvae compared with those in control fish have been observed when whole body as the sample for the real time quantitative assays (Fig. 5K).

Defective HR cell development was also supported by the finding of decreased levels of *ca2* in the PDs near the cloaca, and intestine tissue of *prl*-deficient larvae compared with those of wild-type control fish at the 5 dpf stage (Supplementary Fig. S2A and B). Thus, the down-regulated levels of the specific makers for HR cells in pronephros and NCC cells in the gill epithelium further suggests an impaired Na^+^ absorption in *prl*-null larvae as a whole. All these down-regulated expression might indicate impaired ability to maintain normal Na^+^, K^+^ and Cl^−^ levels in *prl*-deficient larvae, which has been echoed and confirmed by ion content assay (Fig. 2C) and the sodium green staining assay (Fig. 4I,J). The *prl*-deficient larvae showed significantly decreased Na^+^, K^+^ and Cl^−^ levels but normal Ca^2+^ and Mg^2+^ levels at 6 dpf (Fig. 2C). Our ion content assay results were in line with previous studies^21^,^25^.

### Defects in bone formation of *prl*-null zebrafish reared in hypo-osmotic water

**Figure 6:**
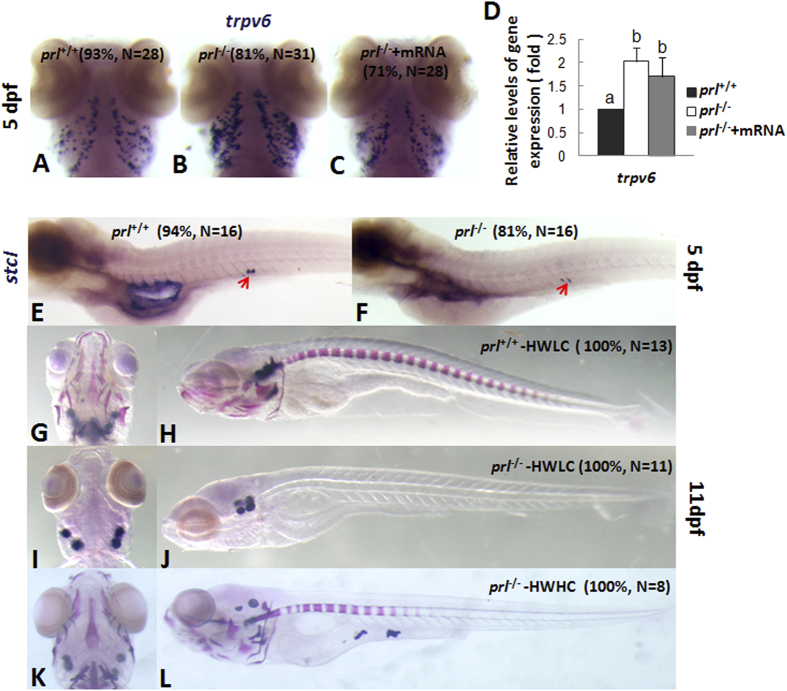
Analyses of Ca^2+^ uptake and bone formation in *prl*-deficient larvae. (**A–C**) Expression patterns of *transient receptor potential family, vanilloid type 6 channel* (*trpv6*) in gills of wild-type larvae (**A**), *prl*-deficient larvae (**B**) *prl*-deficient larvae embryos injected with wild-type *prl* mRNA larvae (**C**) at 5 dpf in regular zebrafish egg water. (**D**) qRT-PCR assay of *trpv6* expression levels in the head tissue from wild-type, *prl*-deficient larvae and *prl*-deficient larvae injected with *prl* mRNA at 5 dpf in regular zebrafish egg water. (**E,F**) Expression patterns of *stanniocalcin 1* (*stc1*) in the corpuscles of Stannius of wild-type (**E**) and *prl*-deficient larvae (**F**) at 5 dpf in regular zebrafish egg water. (**G–L**) Bone pattern assayed with the Alizarin staining for the wild type control larvae (**G,H**), *prl*-deficient larvae (**I,J**) in hypotonic water with low level Ca^2+^ added (salinity: 675 mg IOS/L, Ca^2+^: 71.68 mg/L), and *prl*-deficient larvae (**K,L**) in hypotonic water with high level Ca^2+^ added (salinity: 675 mg IOS/L, Ca^2+^: 143.36 mg/L) at 11 dpf. (**G,I,K**) dorsal views; (**H,J,L**) lateral views. (**a,b**) different letters between two groups mean significant difference (P < 0.05). Dpf, days post-fertilization; qRT-PCR, quantitative real time RT-PCR; IOS, Instant ocean salts. The qRT-PCR result shown here is the representative of the results obtained in two separate experiments. For whole mount *in situ* hybridization, at least 16 embryos/genotype were analyzed in two separated experiments.

The epithelial Ca^2+^ channel *(Trpv6, ECaC*) is critical for epithelial Ca^2+^ uptake and is a specific molecular marker for NaR cells^12^. Enhanced expression levels of *trpv6* in *prl*-deficient larvae was seen via both *in situ* hybridization and qRT-PCR at 5dpf (Fig. 6A–D), while the altered *trpv6* expression could be recovered partially in *prl*-deficient larvae after *prl* mRNA injection (Fig. 6C,D). However, this enhanced expression might not reflect better Ca^2+^ absorption capacities of the *prl*-deficient larvae than those of control fish, as the calcium content wasn’t evaluated in *prl*-deficient larvae at 6 dpf (Fig. 2C). The enhanced *trpv6* expression might be result from an impaired Na^+^-dependent basolateral cation exchanger under the decreased Na^+^ levels^26^. Due to the tiny size of the zebrafish larva at the early stages prior to the death of *prl*-deficient larvae, it is impractical for us to test the levels of plasma Ca^2+^. However, it has been suggested that the expression levels of *stanniocalcin 1* (*stc1*) positively correlated with Ca^2+^ levels in fish serum^27^. Based on our hybridization assays, we observed decreased *stc1* expression levels in *prl*-deficient larvae compared with those of wild-type fish (Fig. 6E,F). This result could actually suggested the decreased Ca^2+^ levels sensed in *prl*-deficient larvae. To confirm this conclusion further, bone staining with Alizarin red was conducted on wild-type, kept in hypotonic water with low calcium level (HWLC), and *prl*-deficient larvae kept in hypotonic water with low and high level of calcium (HWHC). In HWLC, *prl*-deficient larvae showed severe retardation in bone formation compared to wild-type in same water condition at 11 dpf (Fig. 6G–J), while the defect could be rescued effectively in HWHC (Fig. 6K,L). This result proved the bone formation system is still working in *prl*-deficient larvae while the status of Ca^2+^ homeostasis, including up-taking and transportation, could be impaired, even with an enhanced *trpv6* expression levels observed.

## Discussion

The osmoregulatory effects of teleost PRL have been demonstrated by experiments involving hypophysectomy and homologous PRL replacement^8^,^28^. Being a FW species, zebrafish survive FW well through their ability to actively regulate their body osmolarity and maintain the homeostasis of their water content. In our present study, after gene-specific knockout of *prl*, which was proved by *prl* mRNA rescue experiment, while both the phenotype of gas bladders and ion transporter expression could get recovered after synthesized wild-type *prl* mRNA injection (Figs 2 and 4, 5, 6), otherwise these apparently healthy zebrafish with a specific genetic defect for PRL production (*prl*-deficient) lose their ability to survive in FW (Fig. 2A,B), which clearly suggests an osmoregulatory role for zebrafish PRL without any possible risk of physiological interference caused by the surgical procedure removal of the pituitary previously^8^. Furthermore, the fact that *prl*-deficient zebrafish not only survive but thrive in an artificial high salt BW environment (Fig. 2) also strongly suggests that the deficiency is related to a dynamic absorption process engendered in the mutant fish. Consistent with this notion, no significant alterations were observed in *prl*-deficient fish compared with control fis of the expression levels of occludins, claudins, and aquaporin family proteins (Supplementary Fig. S1A-C), which are major components governing the barrier properties of tight junction complexes and of non-ionic compound trans-membrane complexes^29^. In additional, a solution with similar osmolarity created with added mannitol in RSW failed to show any protective effects for the prl-defective larvae (Supplementary Fig. S1D). It would appear, therefore, it has been the passive ion infusion resulting from the high concentrations of ions in BW that can effectively rescue *prl*-deficient fish. This model is supported retrospectively by the observations of the down-regulated expression patterns of ion transporters in the gills and pronephros of both wild-type and rescued *prl*-deficient larvae when raised in BW (Supplementary Fig. S3).

It has been suggested that ionocytes of the branchial epithelia of the gill are essential for maintenance of the systemic salt and water balance in zebrafish. Injected homologous PRL was shown to increase the transcription of *slc12a10.2*, but not of *trpv6* or *slc9a3.2*, in zebrafish gills^14^. However, in our present study, we observed significantly decreased expression levels of *slc12a10.2* in the gills of *prl*-deficient fish compared with wild-type fish at the 6 dpf stage, when mortalities began to be seen (Fig. 4). In addition, although we observed relatively increased levels of the HR cell marker *atp1a1a.5* in gills by *in situ* hybridization, the overall transcription levels of *atp1a1a.5* in *prl*-deficient fish as measured by qRT-PCR were decreased compared with those of the wild-type control fish at the same time, including the transcripts in the gills and the pronephros (Fig. 5). In addition, expression of *slc9a3.2* and *ca2*, two additional apical transporters present in HR cells along with *atp1a1a.5*, was primarily detected solely in the pronephros at these stages, also with decreased levels in *prl*-deficient compared to control fish (Fig. 5, Supplementary Fig. S2), suggesting that functional HR cells exist in the pronephros at this developmental stage. Therefore, even taking into account the suggestion that compensatory regulation of Na^+^ absorption effects might be seen between NCC (*slc12a10.2* expressing) and HR cells (*slc9a3.2* expressing) in zebrafish^21^, the dramatically decreased levels of expression of these proteins arising from both NCC and HR cells together in the *prl*-deficient fish might cause defective Na^+^ absorption (Fig. 4 and 5). Furthermore, it has also been implied that an impaired Cl^−^ homeostasis might be due to decreased NCC cells, which also are thought to take a dominant role in Cl^−^ uptake from ambient environment^22^, as down-regulation of *slc12a3*, whose mammalian ortholog is expressed in the distal collecting tubule (DCT), is important for Na^+^ and Cl^−^ re-absorption^30^,^31^.

It has been reported that zebrafish trpv6 could not be regulated by the recombinant ovine PRL treatment^14^. However, in our present experiments, although elevated expression levels of *trpv6* was observed in *prl*-deficient fish (Fig. 6), defective Ca^2+^ uptake or transportation processes reflected by the defects of the retardation of bone development and decreased levels of *stc1* expression at the 5 dpf stage have been observed (Fig. 6). In addition, the defects of bone formation in *prl* mutant fish could be simply recovered significantly in an elevated Ca^2+^ solution, and most of the *prl*-deficient fish could survive and live healthy to their adulthood without obvious bone defects later on in BW containing elevated Ca^2+^ levels, which suggesting an impairment of Ca^2+^ homeostasis occurred *in vivo*. In consideration of the essential role of Ca^2+^ uptake and transportation for bone development, it’s believed that the enhanced *trpv6* expression seen in *prl*-deficient fish reflects a feedback compensatory response due to the impairment of Ca^2+^ homeostasis in the *prl*-deficient organism, arising from the defective Na^+^/K^+^/Cl^−^ flow and homeostasis status (Figs 4, 5, 6). However, the *prl*-deficient larvae showed no obvious differences in Ca^2+^ content or cartilage development at 6 dpf in EW, when the mortalities had already occurred (Fig. 2). Therefore, it is rational to posit that the Ca^2+^ defect occurred secondary to defective homeostasis of Na^+^, K^+^, and Cl^−^.

Because of its maternally-derived transcripts and maintenance of expression during embryogenesis, PRL has been suggested to be a critical survival factor for organogenesis based on several morpholino knock-down experiments^32^,^33^. Utilizing our newly generated *prl*-deficient zebrafish, especially under the conditions of BW, we are able to obtain offspring from adult *prl*-deficient parents, enabling us to examine this question further. Without maternally derived PRL in F3 embryos obtained from the homozygous mutant parents, we have observed a relatively normal organogenesis process prior to the 5 dpf stage. The level of *foxi3a* expressing ionocyte progenitors is also quite normal in *prl*-deficient embryos (Fig. 3). The specification pathways for ionocyte differentiation, such as into NCC, HR, and NaR cells, seem to be intact as well until 5 dpf. Only the proportions of cell types within the ionocyte pool, and their tissue destinations, were impaired in the *prl*-deficient fish (Figs 4, 5, 6), indicating a critical function for PRL in the fine-tuning of osmoregulation. With the many types of ionocytes remaining in *prl*-deficient larvae and adult fish (Figs 3, 4, 5, 6, and Supplementary Fig. S2), it has been suggested that PRL is therefore not a survival factor for zebrafish ionocytes.

As a freshwater fish, zebrafish inhabit a hypotonic environment and are constantly water loaded and salt depleted through their gill epithelium and skin due to osmotic gradients. To excrete redundant water, zebrafish must produce large volumes of urine. To maintain enough ions for normal osmotic pressure, on one hand, zebrafish have to actively uptake ions from environmental water through their gills, and on the other hand, they must also reabsorb ions through their kidneys by excreting diluted urine^12^,^34^,^35^. The important role of the gills in osmoregulation has been supported by many studies^12^,^29^, whereas the study of the pronephros in zebrafish osmoregulation has been caught less attention compared to those on the gill. During embryogenesis, zebrafish form a simple pronephros comprised of a pair of nephrons, and continue to use this structure over several weeks of larval life. The onset of glomerular filtration of the zebrafish pronephros begins at 2 dpf. However, effective glomerular filtration and salt re-absorption needs to extend to the 4–5 dpf stage, as the presence of mature slit diaphragms and proper renal responses to salt loading occur after the 100 hpf stage^36^,^37^. Consistent with the notion of a critical function of zebrafish *prl* on osmoregulation, expression of both zebrafish *prl* receptors has been observed primarily in both the gills and kidneys (Supplementary Fig. S4). The phenotypes of *prl*-deficient larvae begin to manifest around 5 dpf with mortalities seen after 6 dpf, coincident with the onset of effective renal function for ion reabsorption in healthy zebrafish and the decreased ion contents in *prl*-deficient larvae at same stage (Fig. 2). Together, these observations strongly suggest that impaired renal functions for ion homeostasis might be the primary cause for the death of *prl*-deficient fish.

The involvement of PRL in teleost reproduction has long been known^6^, in part due to the coincidence of increasing plasma PRL of migrating parents adapting to BW or FW during their spawning journey. With the observation that an intact life cycle of *prl*-deficient zebrafish could be accomplished in the BW system, our results have demonstrated that the basal function of PRL in zebrafish is related to osmoregulation. More studies are still needed to fully understand *prl* signaling, in particular to elucidate the mechanisms of its transcriptional regulation of ion transporter molecules.

## Materials and Methods

### Zebrafish maintenance

All zebrafish were kept in a circulated water system with a 14 h light and 10 h dark cycle at 27–28 °C. Fish were kept in four types of water prepared with different doses of Instant Ocean salts (IOS, Coral reef salt, Sunnyvale, CA, Germany). Fresh water (FW) included egg water (60 mg/L IOS) for early stages (<6 dpf) and regular system water (175 mg/L IOS) for juvenile and adult stages. Hyperosmotic brackish water (BW, 5060–5175 mg/L IOS) was prepared by adding 5 g IOS to FW. All the details for the types of the water used in the experiments were listed in Supplementary Table S1. Adult *prl*-deficient zebrafish were maintained only in BW. All animal experiments were conducted in accordance with the Guiding Principles for the Care and Use of Laboratory Animals and were approved by the Institute of Hydrobiology, Chinese Academy of Sciences (Approval ID: IHB 2013724).

### *Prl* gene knockout by TALENs and RNA injection

Paired TALENs were constructed as described^38^ using the Golden Gate TALEN Kit (Addgene, Cambridge, MA, USA). The transcription activator-like (TAL) effector was assembled using the zebrafish *prl1* gene sequence for recognition and binding to the targeting site and the *FokI* gene sequence fused to the TAL effector repeats in the destination vector for DNA cleavage. A restriction site within in the spacer region between the two TALEN arms was used to detect nucleotide change by enzyme digestion. The TALEN plasmids were linearized by *Sac*I and mRNAs were synthesized using the T3 mMESSAGE mMACHINE Kit (Ambion, Austin, TX, USA). Approximately 300–500 pg TALEN mRNAs were microinjected into 1- or 2-cell stage zebrafish embryos, which were then incubated at 28.5 °C. Following hatching, 10–20 embryos were collected for DNA extraction. The target gene region was amplified using the primers: Forward: 5′-GTT TAA GCC CCA CAC CTG GAG T-3′, Reverse: 5′-CGG AAG TCA CGA TAG ACC CGA A-3′, and the polymerase chain reaction (PCR) products were digested by *Xba*I. In this assay, a mutation in the spacer region would result in incomplete digestion of the amplified PCR products. The remainder of the embryos were raised up to adults as F0 and mated with wild-type zebrafish for to generate the F1 generation. The F1 adults were genotyped by enzyme digestion and DNA sequencing of the PCR product amplified from their caudal fin. F1 strains harboring the detected mutations were crossed to each other to obtain the F2 generation, a quarter of homozygous mutants were identified by genotyping. Since the *prl*-deficient fish could live healthy to their adulthood and reproduce normally in BW, the *prl*-deficient homozygous mutants could be then also obtained as the progenies from the mating between the homozygous mutant parents.

For *prl* mRNA injection, full length of zebrafish *prl* cDNA was cloned into pSP64-poly A vector (promega) and the capped mRNA were synthesized using the mMACHINE SP6 Kit (Ambion, AM1340) according to the manufacturer’s instruction. *prl* mRNA was diluted with nuclease-free water to 300 ng/ul and injected into 1- or 2-cell stage embryos.

### Freshwater, brackish water and mannitol added water acclimation experiments

In the rescue experiment, one hundred healthy wild-type or *prl*-deficient homozygous mutant embryos were maintained in FW (1.61–4.29 mOsm/L) or BW (161–167 mOsm/L) respectively, and the survival rates were recorded for 20 days. To investigate whether the rescue effect for *prl-*deficient larvae in brackish water result from preventing water influx, *prl*-deficient homozygous embryos were acclimated to mannitol added water (167 mOsm/L) at the same osmolarity of brackish water (15 g mannitol per liter fresh water).

### RNA isolation and quantitative real time reverse transcription PCR (qRT-PCR)

Zebrafish larvae kept in FW till 5 dpf, head, decapitated body and whole body were collected. Total RNA was extracted using TRIzol (Invitrogen, Carlsbad, CA, USA). RNA templates (5 μg) were used for reverse transcription. cDNAs were synthesized using the RevertAid First Strand cDNA Synthesis Kit (#K1622, Thermo Fisher Scientific, Waltham, MA, USA) with oligo-dT primers. qRT-PCR primers were designed using the National Center for Biotechnology Information (NCBI) primer blast service and are listed in Supplementary Table S2. Each 20-μL amplification reaction contained 10 μL SYBR Green Real-time PCR Master Mix Plus (Toyobo, Osaka, Japan), 0.5 μM each forward and reverse primers, and 1 μL cDNA template. qRT-PCRs were carried out on a BioRad real time system (BioRad Systems, Berkeley, CA, USA). All mRNA levels were calculated as fold expression relative to the housekeeping gene *β-actin1*.

### Whole mount *in situ* hybridization

Whole mount *in situ* hybridization was carried out as described previously^39^,^40^,^41^. Information for all genes assayed via whole mount *in situ* hybridization is listed in Supplementary Table S3.

### Western blotting

Adult zebrafish were anesthetized with 0.2 mg/mL 3-amino benzoic acid ethylester (Tricaine, Sigma-Aldrich, St. Louis, MO, USA) and the pituitaries were dissected for protein extraction as follows: Ten pituitaries were pooled as one sample in 1.5 mL centrifuge tubes that contained 100 μL lysis buffer with 1 mM phenylmethylsulfonyl fluoride (PMSF), and allowed to stand at room temperature (RT) for 10 min. The samples were then sonicated and incubated on ice for another 10 min, followed by centrifugation at 12,000 rpm (4 °C) for 5 min. The supernatants were mixed with an equal volume of 2X sample buffer, and boiled for 5 min. The proteins were separated by 12% sodium dodecyl sulfate-polyacrylamide gel electrophoresis (SDS-PAGE) and transblotted onto a nylon membrane. The primary antibody used for detection was a monoclonal antibody against PRL of common carp^42^. After incubation of the blot with the primary antibody, the membrane was washed in Tris (hydroxymethyl)aminomethane-buffered saline with Tween 20 (TBST) for 4 × 10 min, and incubated with 1:1000 diluted horseradish peroxidase (HRP)-labeled goat anti-mouse IgG at RT for 2 h. A chemiluminescent substrate was applied to the blot for signal detection using a CCD camera-based imager (Alpha Fluorchem Q, Alpha Innotech, San Leandro, CA, USA).

### Bone staining

In order to obtain enough *prl*-deficient and control wild-type larvae at 11 dpf stage for bone staining assay, fertilized eggs were maintained in hypotonic water with lower level of calcium (sanility: 675 mg/L, Ca^2+^: 71.68 mg/L). For testing the rescue effects of the Ca^2+^, a higher level of Ca^2+^ (sanility: 675 mg/L, Ca^2+^: 143.36 mg/L) solution was used. The larvae were collected at the 11 dpf stage, and fixed in 4% paraformaldehyde in phosphate buffered saline (PBS) at 4 °C overnight. The staining procedure was performed according to a previous study^43^. For bone staining by Alizarin, The fixed larvae were gradually dehydrated into 50% ethanol at RT for 10 min. For bone staining by Alizarin, bleaching in 1% H_2_O_2_/0.5% KOH for 10 min and digesting in 1 mg/mL trypsin in 60% saturated sodium borate for 10 min, then staining with freshly prepared 0.04 mg/mL Alizarin solution for 10 min at RT with rocking. Samples were washed briefly in 1% KOH for 4 × 30 s before being photographed with a fluorescent microscope (OLYMPUS-SZX16, Tokyo, Japan).

### Sodium influx analysis

The sodium uptake by ionocytes was visualized using sodium green (S6901, Invitrogen) staining as described^44^. Living larvae at 6 dpf were incubated in 10 μM sodium green in egg water containing 0.1% dimethyl sulfoxide (DMSO) for 1 h at 37 °C. The larvae were anesthetized for photography (OLYMPUS-SZX16).

### Whole body ion content measurement

Fifty zebrafish larvae that had been kept in FW were rinsed in deionized water and pooled as one sample at 6 dpf. After drying out at 56 °C for 6 h, 100 μL HNO_3_ (71%) was added to the samples for digestion at 65 °C overnight. For Na^+^, K^+^, Ca^2+^, and Mg^2+^ measurements, the digested solution was diluted with double-deionized water to a volume of 5 mL and measured using inductively coupled plasma-optical emission spectroscopy (ICP-OES, PerkinElmer-OPTIMA 8000DV, Waltham, MA, USA). For chloride measurements, twenty zebrafish larvae were collected as one sample at 6 dpf. Samples was sonicated in 0.5 mL double-deionized water for 10 min till homogeneous, and the supernatant was collected after centrifuging at 14,000 rpm for 10 min. the chloride contents of the supernatants were measured using chloride colorimetric assay kit (K530-100, Biovision, USA) according to the manufacturer’s instruction.

### Statistical analysis

All of the results are presented as the mean ± S.D. The quantitative experiments were analyzed through one-way ANOVA. Unless otherwise specified, the differences were considered to be significant if the P values were less than 0.05. In this paper, the P values are summarized with the following symbols: *0.01 < P ≤ 0.05; ***P ≤ 0.01.

## Additional Information

**How to cite this article**: Shu, Y. *et al.* The basal function of teleost prolactin as a key regulator on ion uptake identified with zebrafish knockout models. *Sci. Rep.* **6**, 18597; doi: 10.1038/srep18597 (2016).

## Supplementary Material

Supplementary Information

## References

[b1] Riddle O., Bates R. W. & Dykshorn S. W. The preparation, identification and assay of prolactin - A hormone of the anterior pituitary. Am J Physiol 105, 191–216 (1933).

[b2] Ormandy C. J. *et al.* Null mutation of the prolactin receptor gene produces multiple reproductive defects in the mouse. Genes & development 11, 167–178 (1997). 9009200 10.1101/gad.11.2.167

[b3] Wennbo H. *et al.* Activation of the prolactin receptor but not the growth hormone receptor is important for induction of mammary tumors in transgenic mice. The Journal of clinical investigation 100, 2744–2751 (1997). 9389738 10.1172/JCI119820 PMC508478

[b4] Ben-Jonathan N., LaPensee C. R. & LaPensee E. W. What can we learn from rodents about prolactin in humans? Endocr Rev 29, 1–41 (2008). 18057139 10.1210/er.2007-0017 PMC2244934

[b5] Horseman N. D. & Gregerson K. A. Prolactin actions. J Mol Endocrinol 52, R95–106 (2014). 10.1530/JME-13-0220 24130130

[b6] Whittington C. M. & Wilson A. B. The role of prolactin in fish reproduction. Gen Comp Endocrinol 191, 123–136 (2013). 23791758 10.1016/j.ygcen.2013.05.027

[b7] Manzon L. A. The role of prolactin in fish osmoregulation: a review. Gen Comp Endocrinol 125, 291–310 (2002). 11884075 10.1006/gcen.2001.7746

[b8] Pickford G. E. & Phillips J. G. Prolactin, a factor in promoting survival of hypophysectomized killifish in fresh water. Science 130, 454–455 (1959). 13675773 10.1126/science.130.3373.454

[b9] Madsen S. S., Jensen M. K., Nohr J. & Kristiansen K. Expression of Na+-K+-ATPase in the brown trout, Salmo trutta: *In vivo* modulation by hormones and seawater. Am J Physiol-Reg I 269, R1339–R1345 (1995). 10.1152/ajpregu.1995.269.6.R1339 8594935

[b10] Seale A. P. *et al.* Effects of salinity and prolactin on gene transcript levels of ion transporters, ion pumps and prolactin receptors in Mozambique tilapia intestine. Gen Comp Endocrinol 206, 146–154 (2014). 25088575 10.1016/j.ygcen.2014.07.020

[b11] Sakamoto T. & McCormick S. D. Prolactin and growth hormone in fish osmoregulation. Gen Comp Endocrinol 147, 24–30 (2006) 16406056 10.1016/j.ygcen.2005.10.008

[b12] Hwang P. P. & Chou M. Y. Zebrafish as an animal model to study ion homeostasis. Pflug Arch Eur J Phy 465, 1233–1247 (2013). 10.1007/s00424-013-1269-1 PMC3745619 23568368

[b13] Huang X. G. *et al.* Discovery of a Novel Prolactin in Non-Mammalian Vertebrates: Evolutionary Perspectives and Its Involvement in Teleost Retina Development. Plos One 4, e6163 (2009). 19584915 10.1371/journal.pone.0006163 PMC2702173

[b14] Breves J. P., Serizier S. B., Goffin V., McCormick S. D. & Karlstrom R. O. Prolactin regulates transcription of the ion uptake Na+/Cl- cotransporter (ncc) gene in zebrafish gill. Mol Cell Endocrinol 369, 98–106 (2013). 23395804 10.1016/j.mce.2013.01.021 PMC3664226

[b15] Zhu Y., Song D., Tran N. T. & Nguyen N. The effects of the members of growth hormone family knockdown in zebrafish development. Gen Comp Endocrinol 150, 395–404 (2007). 17141235 10.1016/j.ygcen.2006.10.009

[b16] Chasiotis H. & Kelly S. P. Occludin immunolocalization and protein expression in goldfish. J Exp Biol 211, 1524–1534 (2008). 18456879 10.1242/jeb.014894

[b17] Cerda J. & Finn R. N. Piscine Aquaporins: An Overview of Recent Advances. J Exp Zool Part A 313A, 623–650 (2010). 10.1002/jez.634 20717996

[b18] Hsiao C. D. *et al.* A positive regulatory loop between foxi3a and foxi3b is essential for specification and differentiation of zebrafish epidermal ionocytes. Plos One 2, e302 (2007). 17375188 10.1371/journal.pone.0000302 PMC1810426

[b19] Chang W. J. & Hwang P. P. Development of Zebrafish Epidermis. Birth Defects Research Part C-Embryo Today 93, 205–214 (2011). 10.1002/bdrc.20215 21932430

[b20] Kumai Y., Kwong R. W. & Perry S. F. The role of cAMP-mediated intracellular signaling in regulating Na+ uptake in zebrafish larvae. American journal of physiology. Regulatory, integrative and comparative physiology 306, R51–60 (2014). 10.1152/ajpregu.00317.2013 PMC3921305 24259461

[b21] Chang W. J. *et al.* Compensatory regulation of Na+ absorption by Na+/H+ exchanger and Na+-Cl- cotransporter in zebrafish (Danio rerio). Front Zool 10 (2013). 10.1186/1742-9994-10-46 PMC3750650 23924428

[b22] Wang Y. F., Tseng Y. C., Yan J. J., Hiroi J. & Hwang P. P. Role of SLC12A10.2, a Na-Cl cotransporter-like protein, in a Cl uptake mechanism in zebrafish (Danio rerio). American journal of physiology. Regulatory, integrative and comparative physiology 296, R1650–1660 (2009). 10.1152/ajpregu.00119.2009 19279294

[b23] Liao B. K., Chen R. D. & Hwang P. P. Expression regulation of Na+-K+-ATPase alpha1-subunit subtypes in zebrafish gill ionocytes. American journal of physiology. Regulatory, integrative and comparative physiology 296, R1897–1906 (2009). 10.1152/ajpregu.00029.2009 19386990

[b24] Mobasheri A. *et al.* Na+, K+-ATPase isozyme diversity; Comparative biochemistry and physiological implications of novel functional interactions. Bioscience Rep 20, 51–91 (2000). 10.1023/a:1005580332144 10965965

[b25] Kwong R. W. & Perry S. F. The tight junction protein claudin-b regulates epithelial permeability and sodium handling in larval zebrafish, Danio rerio. American journal of physiology. Regulatory, integrative and comparative physiology 304, R504–513 (2013). 10.1152/ajpregu.00385.2012 PMC3627946 23364531

[b26] Despa S. & Bers D. M. Na(+) transport in the normal and failing heart - remember the balance. Journal of molecular and cellular cardiology 61, 2–10 (2013). 23608603 10.1016/j.yjmcc.2013.04.011 PMC3720717

[b27] Tseng D. Y. *et al.* Effects of stanniocalcin 1 on calcium uptake in zebrafish (Danio rerio) embryo. American journal of physiology. Regulatory, integrative and comparative physiology 296, R549–557 (2009). 10.1152/ajpregu.90742.2008 19073903

[b28] Jackson L. F., McCormick S. D., Madsen S. S., Swanson P. & Sullivan C. V. Osmoregulatory effects of hypophysectomy and homologous prolactin replacement in hybrid striped bass. Comp Biochem Phys B 140, 211–218 (2005). 10.1016/j.cbpc.2004.10.004 15649768

[b29] Breves J. P., McCormick S. D. & Karlstrom R. O. Prolactin and teleost ionocytes: New insights into cellular and molecular targets of prolactin in vertebrate epithelia. Gen Comp Endocr 203, 21–28 (2014). 24434597 10.1016/j.ygcen.2013.12.014 PMC4096611

[b30] Schultheis P. J. *et al.* Phenotype Resembling Gitelman’s Syndrome in Mice Lacking the Apical Na+-Cl- Cotransporter of the Distal Convoluted Tubule. Journal of Biological Chemistry 273, 29150–29155 (1998). 9786924 10.1074/jbc.273.44.29150

[b31] Nicolet-Barousse L. *et al.* Inactivation of the Na-Cl co-transporter (NCC) gene is associated with high BMD through both renal and bone mechanisms: analysis of patients with Gitelman syndrome and Ncc null mice. Journal of bone and mineral research : the official journal of the American Society for Bone and Mineral Research 20, 799–808 (2005). 10.1359/JBMR.041238 15824853

[b32] Nguyen N., Stellwag E. J. & Zhu Y. Prolactin-dependent modulation of organogenesis in the vertebrate: Recent discoveries in zebrafish. Comp Biochem Phys C 148, 370–380 (2008). 10.1016/j.cbpc.2008.05.010 18593647

[b33] Nguyen N. & Zhu Y. Prolactin functions as a survival factor during zebrafish embryogenesis. Comp Biochem Phys A 153, 88–93 (2009). 10.1016/j.cbpa.2008.10.019 19032987

[b34] Evans D. H. Teleost fish osmoregulation: what have we learned since August Krogh, Homer Smith, and Ancel Keys. American journal of physiology. Regulatory, integrative and comparative physiology 295, R704–713 (2008). 10.1152/ajpregu.90337.2008 18525009

[b35] Smith H. W. Water regulation and its evolution in the fishes. Q Rev Biol 7, 1–26 (1932).

[b36] Hentschel D. M. *et al.* Rapid screening of glomerular slit diaphragm integrity in larval zebrafish. Am J Physiol-Renal 293, F1746–F1750 (2007). 10.1152/ajprenal.00009.2007 17699558

[b37] Rider S. A. *et al.* Techniques for the *in vivo* assessment of cardio-renal function in zebrafish (Danio rerio) larvae. J Physiol-London 590, 1803–1809 (2012). 22331420 10.1113/jphysiol.2011.224352 PMC3573304

[b38] Cermak T. *et al.* Efficient design and assembly of custom TALEN and other TAL effector-based constructs for DNA targeting. Nucleic acids research 39, e82 (2011). 21493687 10.1093/nar/gkr218 PMC3130291

[b39] Moens C. Whole mount RNA *in situ* hybridization on zebrafish embryos: hybridization. CSH protocols 2008, pdb prot5037 (2008). 10.1101/pdb.prot5037 21356894

[b40] Moens C. Whole mount RNA *in situ* hybridization on zebrafish embryos: mounting. CSH protocols 2008, pdb prot5038 (2008). 10.1101/pdb.prot5038 21356895

[b41] Moens C. Whole mount RNA *in situ* hybridization on zebrafish embryos: probe synthesis. CSH protocols 2008, pdb prot5036 (2008). 10.1101/pdb.prot5036 21356893

[b42] Xu Z. W. *et al.* Production, characterization, and applications of mouse monoclonal antibodies against gonadotropin, somatolactin, and prolactin from common carp (Cyprinus carpio). Gen Comp Endocr 167, 373–378 (2010). 19854193 10.1016/j.ygcen.2009.10.005

[b43] Walker M. B. & Kimmel C. B. A two-color acid-free cartilage and bone stain for zebrafish larvae. Biotechnic & histochemistry : official publication of the Biological Stain Commission 82, 23–28 (2007). 17510811 10.1080/10520290701333558

[b44] Esaki M. *et al.* Visualization in zebrafish larvae of Na+ uptake in mitochondria-rich cells whose differentiation is dependent on foxi3a. AJP: Regulatory, Integrative and Comparative Physiology 292, R470–R480 (2006). 10.1152/ajpregu.00200.2006 16946087

