# Supplementary material for: The basal function of teleost prolactin as a key regulator on ion uptake identified with zebrafish knockout models
Source: Sci Rep. 2016 Jan 4;6:18597. doi: 10.1038/srep18597 (PMC4698586; doi:10.1038/srep18597)
Supplement: Supplementary Information [file srep18597-s1.doc]

**The basal function of teleost prolactin as a key regulator on ion uptake identified with zebrafish knockout models**

Yuqin Shu^1,2^, Qiyong Lou^1^, Ziru Dai^3^, Xiangyan Dai^1^, Gang Zhai^1^, Jiangyan He^1^, Wei Hu^1^,*, and Zhan Yin^1^,*

*^1^Key Laboratory of Aquatic Biodiversity and Conservation of the Chinese Academy of Sciences, Institute of Hydrobiology, Chinese Academy of Sciences, Wuhan, Hubei, China, 430072.* *^2^University of the Chinese Academy of Sciences, Beijing, China. ^3^Key Laboratory of Molecular Biophysics of Minstry of Education, College of Life Science and Technology, Center for Human Genome Research, Huazhong University of Science and Technology, Wuhan, Hubei 430074, P. R. China.*

**Supplementary Figure S1 to S5**

**Supplementary Figure S1**

**Supplementary Figure S1.** No significant effects on water permeability caused in *prl*-deficient fish. A-B) No significant alteration of transcriptional expression levels of tight-junction and aquaporin proteins in *prl*-deficient larvae assayed via qRT-PCR. A) Comparison of transcriptional expression levels of aquaporins 1a.1 (*aqp1a.1*) and 3a (*aqp3a*), and occludins A and B in *prl*-deficient larvae at 5 dpf in regular culture medium with those of wild-type control fish. B) Comparison of transcriptional expression levels of claudins 2, 7, 8, 11, 12, and 19 in *prl*-deficient larvae at 5 dpf in regular culture medium with those of wild-type control fish. C) Comparison of transcriptional expression levels of claudins A, B, C, E, F, G, I, and J in *prl*-deficient larvae at 5 dpf in regular culture medium with those of wild-type control fish. D) *Prl*-deficient fish fail to survive in the mannitol added water (167 mOsm/L) at the same osmolarity of brackish water (15 g mannitol per liter fresh water). qRT-PCR, quantitative real time reverse transcription polymerase chain reaction; dpf, days post-fertilization.

**Supplementary Figure S2**

**
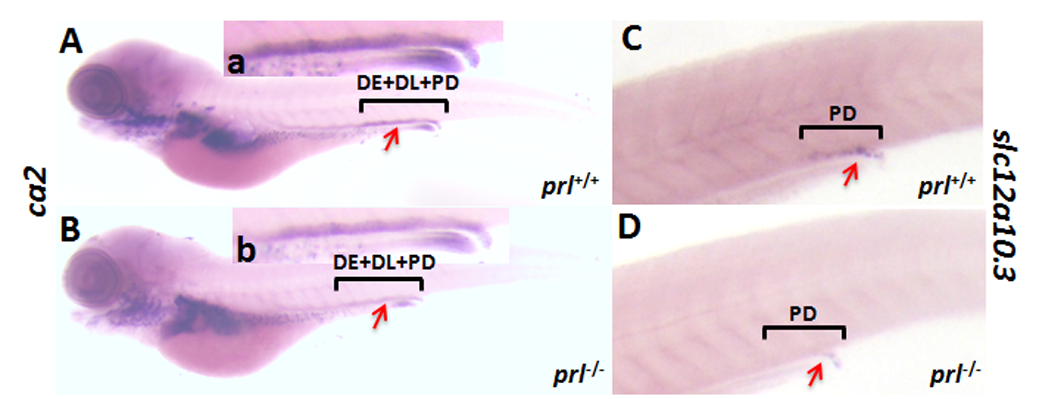
**

**Supplementary Figure S2.** Decreased transcriptional expression of apical transporters in the pronephric duct of *prl*-deficient larvae. Expression patterns were assayed with whole mount *in situ* hybridization. A, B) Decreased expression of carbonic anhydrase II (*ca2*) in the pronephric ducts of *prl*-deficient larvae (B) compared with those of wild-type control fish (A) at 5 dpf in regular culture medium. C, D) Decreased expression of solute carrier family 12, member 10, tandem duplicate 3 (*slc1210.3*) in the pronephric ducts of *prl*-deficient larvae (B) compared with those of wild-type control fish (A) at 5 dpf in regular culture medium. Dpf, days post-fertilization.

**Supplementary Figure S3**

**
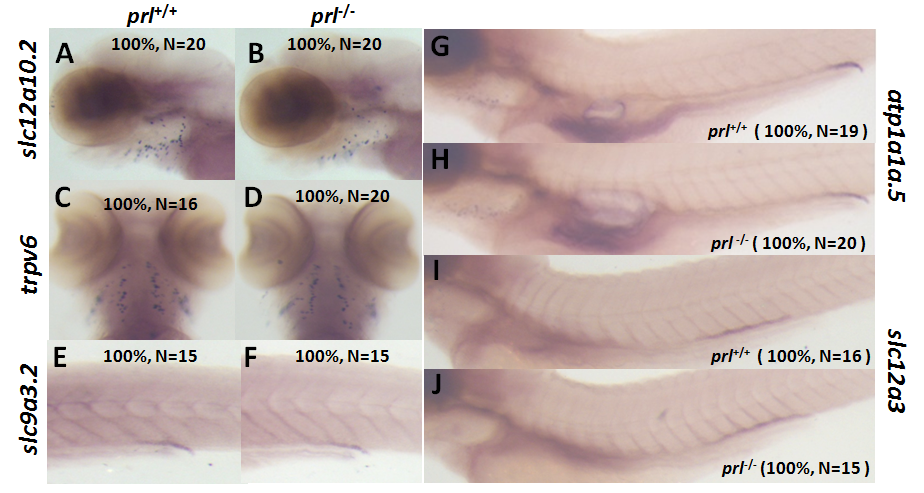
**

**Supplementary Figure S3.** Down-regulation of ion transporter expression patterns of both wild-type and *prl*-deficient larvae at 5 dpf in BW. A-D) Whole mount *in situ* hybridization assay of *slc12a10.2* (A, B) and *trpv6* (C, D) expression in gills of wild-type (A, C) and *prl*^-/-^ (B, D) larvae at 5 dpf in brackish water. E-J) Whole mount *in situ* hybridization assay of *slc9a3.2* (E, F), *atp1a1a.5* (G, H) and *slc12a3* (I, J) expression in pronephric ducts of wild-type (E, G and I) and *prl*^-/-^ (F, H and J) larvae at 5 dpf in BW. Dpf, days post-fertilization.

**Supplementary Figure S4**

**
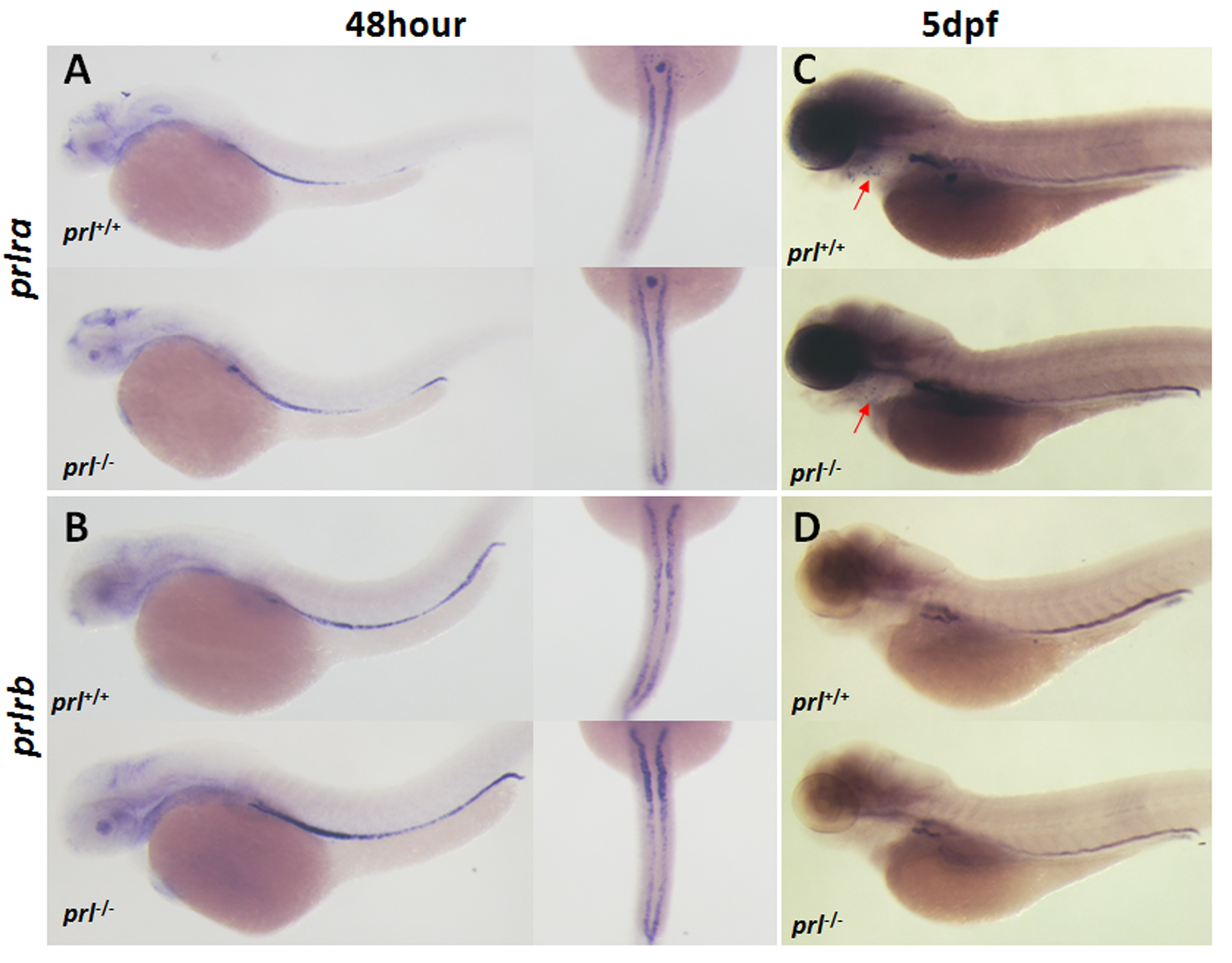
**

**Supplementary Figure S4.** Transcriptional expression patterns of prolactin receptors in early developmental stages as visualized by whole mount *in situ* hybridization analysis. A-B) Expression patterns of prolactin receptors a (*prlra,* A) and b (*prlrb,* B) at 48 hpf. C-D) Expression patterns of *prlra* (C) and *prlrb* (D) at 5 dpf. Hpf, hours post-fertilization; and dpf, days post-fertilization.

**Supplementary Table S1 to S3**

**Supplementary Table S1** **The concentration of the major ions of four types’ water**

| **Ion (mM)** | **Na^+^** | **K^+^** | **Ca^2+^** | **Mg^2+^** | **Cl^-^** | **Salinity**  **(mg/L)** | **Osmolarity**  **(mOsmol/L)** |
| --- | --- | --- | --- | --- | --- | --- | --- |
| **EW** | **0.6906** | **0.0186** | **0.0295** | **0.0819** | **1.1408** | **60** | **1.9614** |
| **RSW** | **0.8152** | **0.0909** | **1.5473** | **0.3999** | **0.9299** | **175** | **3.7832** |
| **EW+IOS** | **57.5463** | **1.5467** | **2.4598** | **6.8278** | **95.0663** | **5060** | **163.4469** |
| **RSW+IOS** | **58.3615** | **1.6376** | **4.0071** | **7.2277** | **95.9962** | **5175** | **167.2301** |
| **HWLC** | **6.5008** | **0.2437** | **1.7920** | **1.0745** | **10.3225** | **675** | **19.9335** |
| **HWHC** | **4.1929** | **0.1817** | **3.5840** | **0.8007** | **10.3479** | **675** | **19.1072** |

**EW**: eggater (≤6 dpf)

**RSW**: regular system water (>6 dpf)

**EW+IOS**: egg water added 5g Instant Ocean salts per liter(≤6 dpf)

**RSW+IOS**: regular system water added 5g instant Ocean salts per liter (> 6 dpf)

**HWLC:** regular system water with low level of calcium added 0.5g instant Ocean salts per liter (71.68 mg/L calcium)

**HWHC**: regular system water with high level of calcium added 0.29g Instant Ocean salts and 0.21g CaCl2 per liter (143.36 mg/L calcium)

EW and RSW were identified as freshwater (FW), EW+IOS and RSW+IOS were identified as brackish water (BW)

**Supplementary Table S2 Primers used for Q-PCR.**

| genes | primers sequence |  |
| --- | --- | --- |
| *β-actin* | F: 5'-GCC ACC TTA AAT GGC CTA GCA-3' |  |
|  | R: 5'-GCC ATA CAG AGC AGA AGC CA-3' |  |
| *foxi3a* | F: 5'-GCGATGGCAATACATGGAGC-3' |  |
|  | R: 5'-TTG CCT TTT CCT GGA TCG CT-3' |  |
| *foxi3b* | F: 5'- AGA TGA TCC AGG GAA GGG CA-3' |  |
|  | R: 5'- TCT TGC GTC TGA AGT TGC CA-3' |  |
| *atp1b1b* | F: 5'- AGA CGA CGG AGG ATG GAA GA-3' |  |
|  | R: 5'- AGA TCT TTG CCC AAC TCC CG-3' |  |
| *ca2* | F: 5'- AGG ACG CAG TTG ATA AGC CT-3' |  |
|  | R: 5'- TGG ACT TGA TAG CAT CCA TA-3' |  |
| *slc12a10.2* | F: 5'-GAC CCA AGG TGG AGA GGA CG-3' |  |
|  | R: 5'-CAG TTG ATA CCG ATA CTC AGC-3' |  |
| *slc12a3* | F: 5'-CGA TGA TGGC GGT TTG ACA C-3' |  |
|  | R: 5'-TGA AAC CCA GAC GGA ACC TG-3' |  |
| *slc9a3.2* | F: 5'-GCG AAA CCC ACC CTG GCA AAC-3' |  |
|  | R: 5'-GGC GAA GGA GTC TGT GGA GCG-3' |  |
| *atp1a1a.5* | F: 5'-GTG GTT CTG TGC CTT CCC AT-3' |  |
|  | R: 5'-CGG GTG TTC ATT TTG ATG TT-3' |  |
| *trpv6* | F: 5'-TGG CTC AGG ATG CAG AAC AG-3' |  |
|  | R: 5'-CAA GTG CTG GAA GAC GTT GC-3' |  |
| *aqp1a.1* | F: 5'-CCA TCA GCT TCA CGG GAT GT-3' |  |
|  | R: 5'-CCA GTA GAC CCA GTG GTT GG-3' |  |
| *aqp3a* | F: 5'-GAT GGG TTG GCA GAA AAG CG-3' |  |
|  | R: 5'-ACC ACA GCC AAA CAT CAC CA-3' |  |
| *18S* | F: 5'-GGC GGC GTT ATT CCC ATG ACC-3' |  |
|  | R: 5'-GGT GGT GCC CTT CCG TCA ATT C-3' |  |
| *occludinA* | F: 5'-GGG TCT GCT GGC TGA CTA TC-3' |  |
|  | R: 5'-GAA TCT CCA CGG GAC TTT CA-3' |  |
| *occludinB* | F: 5'-GAC CAT TAA GGA TGG CCT CA-3' |  |
|  | R: 5'-GCT GAG CAG CAC TGA CTT TG-3' |  |
| *claudinA* | F: 5'-TAA TTG CCC TCC ACA AGA CC-3' |  |
|  | R: 5'-AAA GCT GTC CAG CAT CCA AT-3' |  |
| *claudinB* | F: 5'-AGA CAG CGG AAA ATA CAC AGC-3' |  |
|  | R: 5'-TGA GCC TCA ATG TCC AAC AA-3' |  |
| *claudinC* | F: 5'-GTA CCC TCC GCA AAG TCG TA-3' |  |
|  | R: 5'-CTT TCA AGG AAA GAC TGA CAG C-3' |  |
| *claudinD* | F: 5'-GGG TCG CGC TTA TTC TGT TA-3' |  |
|  | R: 5'-TTC CTA CAC AAA CAG GAG ACG AT-3' |  |
| *claudinE* | F: 5'-CTG CCC TCC AAA AGA TGA AA-3' |  |
|  | R: 5'-TGG CAA GTC TTA GAA CGA AAG A-3' |  |
| *claudinF* | F: 5'-ACT GAT CGG ATT CAT GGG CA-3' |  |
|  | R: 5'-GCC TCC CAG AAT GAG CAC TT-3' |  |
| *claudinG* | F: 5'-GGG TCG GTA TAC ACC AGC TT-3' |  |
|  | R: 5'-TCT GCT TTA CAA AGA CGA TCT CA-3' |  |
| *claudinH* | F: 5'-GAA TGG GCT ATT CTG CTC CA-3' |  |
|  | R: 5'-TCA CCC TTT TCA TCC GTC TT-3' |  |
| *claudinI* | F: 5'-GAG CCG CCA AAT ACT ACA GC-3' |  |
|  | R: 5'-TTC GCT ACC TTA GAC GGG TTA-3' |  |
| *claudinJ* | F: 5'-TCT GGC ACT CAC AGT GGC TA-3' |  |
|  | R: 5'-CAG CCC ATA TGA ATA GTT TAC CC-3' |  |
| *claudin2* | F: 5'-CTG TGG GAA AAC ACA GGC AC-3' |  |
|  | R: 5'-AGG GGA GAA GAG TGG CTA CC-3' |  |
| *claudin7* | F: 5'-CTT GCT CAA AGG GTC AGT CA-3' |  |
|  | R: 5'-GTC CTT TCC AGC TCG TGA AC-3' |  |
| *claudin8* | F: 5'-CAT AAT CGG CCT CAT CGG GG-3' |  |
|  | R: 5'-AGC AGT TCA TCC ACA GTC CC-3' |  |
| *claudin11* | F: 5'-CTG TAC ATA CGG GTC GCA CA-3' |  |
|  | R: 5'-ACG ACA CGC CTG GAT GTA AG-3' |  |
| *claudin12* | F: 5'-ATG ACC CAA AGC CTC TCT GC-3' |  |
|  | R: 5'-GGT AAC CCT GCA TTG GGA CA-3' |  |
| *claudin19* | F: 5'-AAG TGC ACA AAG GTG GGT GA-3' |  |
|  | R: 5'-TCG AAG AAG TGG GCA GAC AC-3' |  |
| qRT-PCR = quantitative real time reverse transcription polymerase chain reaction | | |

**Supplementary Table S3.** Gene fragment probes for whole mount *in situ* hybridization.

| Gene Name | GenBank Accession Number | Region |
| --- | --- | --- |
| *slc12a10.2* | NM_001045001.1 | nt 621–2178 bp |
| *slc9a3.2* | NM_001113479.1 | nt 1879–2691 bp |
| *trpv6* | NM_001001849.1 | nt 1759–2221 bp |
| *atp1a1a.5* | NM_178099.2 | nt 1892–3554 bp |
| *slc12a10.3* | NM_001135131.1 | nt 2181–2496 bp |
| *slc12a3* | NM_001045080.1 | nt 587–898 bp |
| *foxi3a* | NM_198917.2 | nt 868–1179 bp |
| *foxi3b* | NM_198918.1 | nt 738–1083 bp |
| *ca2* | NM_199215.1 | nt 364–726 bp |
| *atp1b1b* | NM_131671.1 | nt 558–955 bp |
| *prlra* | NM_001128677.1 | nt 1318–1918 bp |
| *prlrb* | NM_001113500.1 | nt 171–956 bp |
| *stc1* | NM_001045457.1 | nt 477–801 bp |
